# Ultrasensitive nucleic acid detection based on phosphorothioated hairpin-assisted isothermal amplification

**DOI:** 10.1038/s41598-021-87948-8

**Published:** 2021-04-16

**Authors:** Yujin Jung, Jayeon Song, Hyun Gyu Park

**Affiliations:** Department of Chemical and Biomolecular Engineering (BK 21+ Program), KAIST, Daehak-ro 291, Yuseong-gu, Daejeon, 34141 Republic of Korea

**Keywords:** Assay systems, Health care, DNA, Target identification, Target validation, Diseases

## Abstract

Herein, we describe a phosphorothioated hairpin-assisted isothermal amplification (PHAmp) method for detection of a target nucleic acid. The hairpin probe (HP) is designed to contain a 5′ phosphorothioate (PS)-modified overhang, a target recognition site, and a 3′ self-priming (SP) region. Upon binding to the target nucleic acid, the HP opens and the SP region is rearranged to serve as a primer. The subsequent process of strand displacement DNA synthesis recycles the bound target to open another HP and produces an extended HP (EP) with a PS-DNA/DNA duplex at the end, which would be readily denatured due to its reduced thermal stability. The trigger then binds to the denatured 3′ end of the EP and is extended, producing an intermediate double-stranded (ds) DNA product (IP). The trigger also binds to the denatured 3′ end of the IP, and its extension produces the final dsDNA product along with concomitant displacement and recycling of EP. By monitoring the dsDNA products, the target nucleic acid can be identified down to 0.29 fM with a wide dynamic range from 1 nM to 1 fM yielding an excellent specificity to discriminate even a single base-mismatched target. The unique design principle could provide new insights into the development of novel isothermal amplification methods for nucleic acid detection**.**

## Introduction

During the past few decades, strategies based on isothermal amplification have shown great promise as an alternative to conventional PCR-based amplification methods due to their intrinsic advantages, which include low hardware dependence (eliminating the need for a thermocycling machine), quick amplification, and simplicity of the reaction. Since these features are critical to realize point-of-care (POC) or on-site molecular diagnostics in resource-limited settings^1–5^, various types of isothermal amplification methods, including strand displacement amplification (SDA)^6,7^, nucleic acid sequence-based amplification (NASBA)^8,9^, rolling-circle amplification (RCA)^10–12^, helicase-dependent amplification (HDA)^13,14^, recombinase polymerase amplification (RPA)^15,16^, exponential amplification reaction (EXPAR)^17,18^, isothermal chain amplification (ICA)^19,20^, loop-mediated amplification (LAMP)^21,22^, and so on^23–25^ have been extensively developed.

Of these, LAMP technology has particularly attracted great attention owing to its very high amplification efficiency achieved by only a single DNA polymerase and has shown its wide utility in numerous applications of molecular diagnostics^26,27^. In LAMP, the use of foldback structured primers and their subsequent extension quite simplify the amplification step by eliminating the need for a complex combination of enzymes or proteins. The requirement for multiple exogenous primers, however, could increase the complexity of probe design and the cumbersomeness of the procedure^28^. Moreover, the relatively high operating temperature of LAMP (60–65 °C), when compared to several other methods that operate at lower temperatures around 40 °C^6,9,10,14,15^, potentially increases both device complexity and power consumption. Therefore, there is a great incentive existing to develop a more advanced isothermal method that would resolve the above-mentioned limitations while preserving the high amplification efficiency.

Based on this background, we herein describe a novel phosphorothioated hairpin-assisted isothermal amplification (PHAmp) technology capable of detecting target nucleic acids under isothermal conditions, which relies on the recent finding that phosphorothioate (PS)-modified DNA destabilizes the base stacking interaction within the double-helical structure and consequently reduces the melting temperature (Tm) of the PS-DNA/DNA duplex formed with the complementary DNA strand^29,30^. By taking advantage of this behavior, we designed a hairpin probe (HP) that consists of a 5′ PS-DNA overhang, a target recognition site, and a 3′ self-priming (SP) region and verified that the PHAmp reaction is able to reliably identify target nucleic acid in a quite simplified manner requiring using only two simple DNA probes and a single enzyme.

## Results and discussion

### Design and working principle of the PHAmp reaction

**Figure 1:**
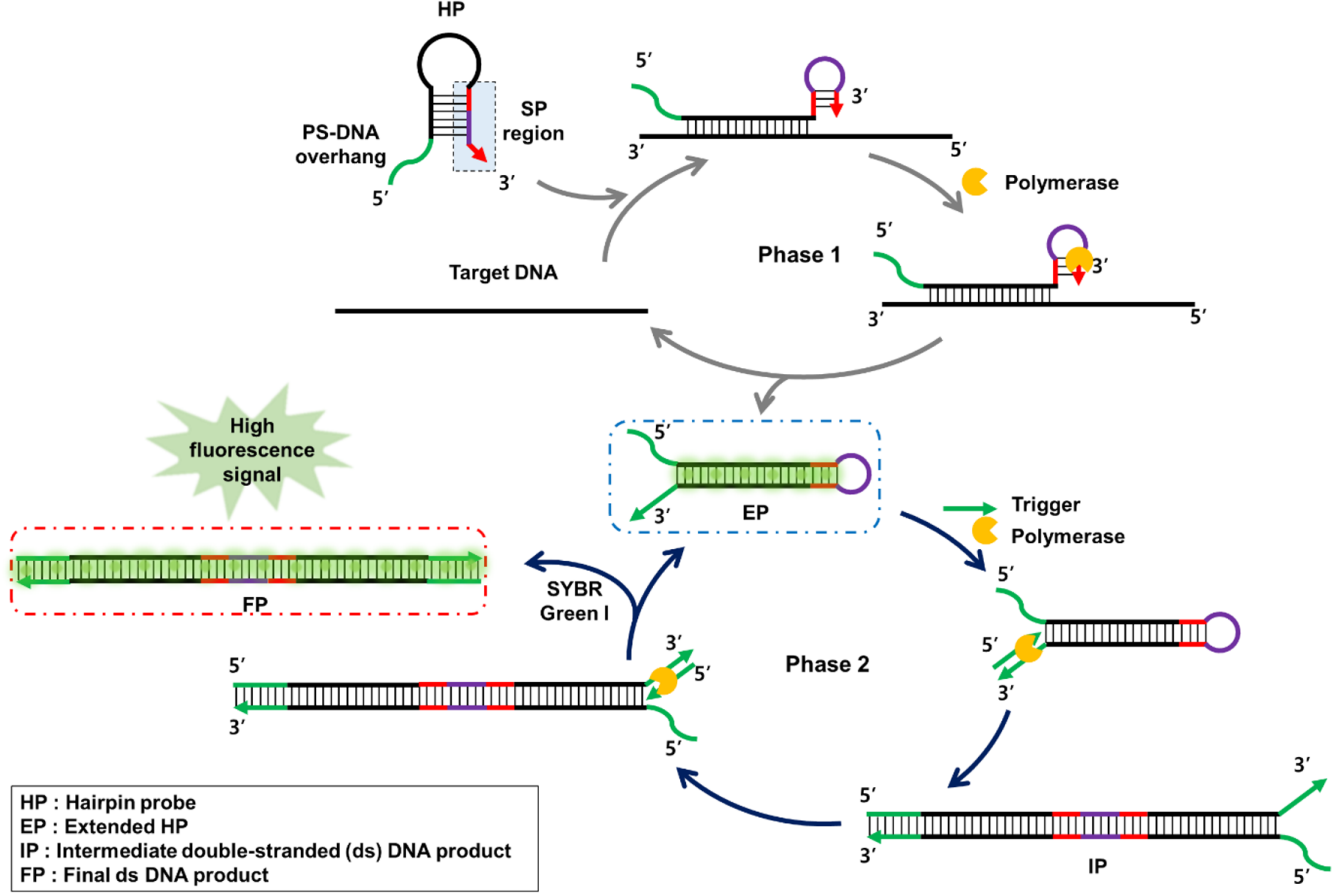
Schematic illustration of the phosphorothioated hairpin-assisted isothermal amplification (PHAmp) for detection of a target nucleic acid. This figure was created using PowerPoint Professional Plus 2016 (https://www.microsoft.com/ko-kr/microsoft-365/powerpoint).

Figure 1 illustrates the working principle of the PHAmp reaction. The key component that actuates the PHAmp reaction is the HP consisting of three functional domains: PS-DNA at the 5′ overhang, a target recognition site in the loop and stem region, and the SP region along the 3′ end. In principle, the PHAmp reaction can be divided into two reaction phases. Phase 1 accomplishes target recognition, target recycling, and production of extended HP (EP), whereas phase 2 produces an intermediate double-stranded (ds) DNA product (IP) and final dsDNA product (FP).

In the absence of target DNA, the HP retains its initial structure, and the SP region is kept partially blocked by the stem-loop structure. Therefore, the PHAmp reaction does not proceed. In the presence of target DNA, however, it binds to the target recognition site within HP to open the HP, consequently promoting a foldback reaction that rearranges the SP region to form a self-priming structure at the 3′ end. The following strand displacement DNA synthesis then produces EP whose stem end is composed of a PS-DNA/DNA duplex and concomitantly recycles target nucleic acid to initiate another phase 1 reaction by binding to another free HP. The EP then enters and initiates the phase 2 reaction. Due to the reduced thermal stability of the stem end of the EP, the EP would be more readily denatured at the end under the reaction temperature (37 °C) during dsDNA breathing phenomenon, which allows the trigger to anneal to the 3′ end of EP^31,32^. Extension of the trigger annealed to the EP produces an IP, which also contains a PS-DNA/DNA duplex at one end. The trigger also binds to the denatured 3′ DNA of the IP in the same manner and is extended to produce an FP without any PS-DNA modification, simultaneously displacing and recycling the EP to initiate another phase 2 reaction. The combination of these two reaction phases would produce a large number of EPs and FPs, which can be monitored in real-time via duplex-specific SYBR Green I staining.

### Feasibility of the PHAmp reaction

**Figure 2:**
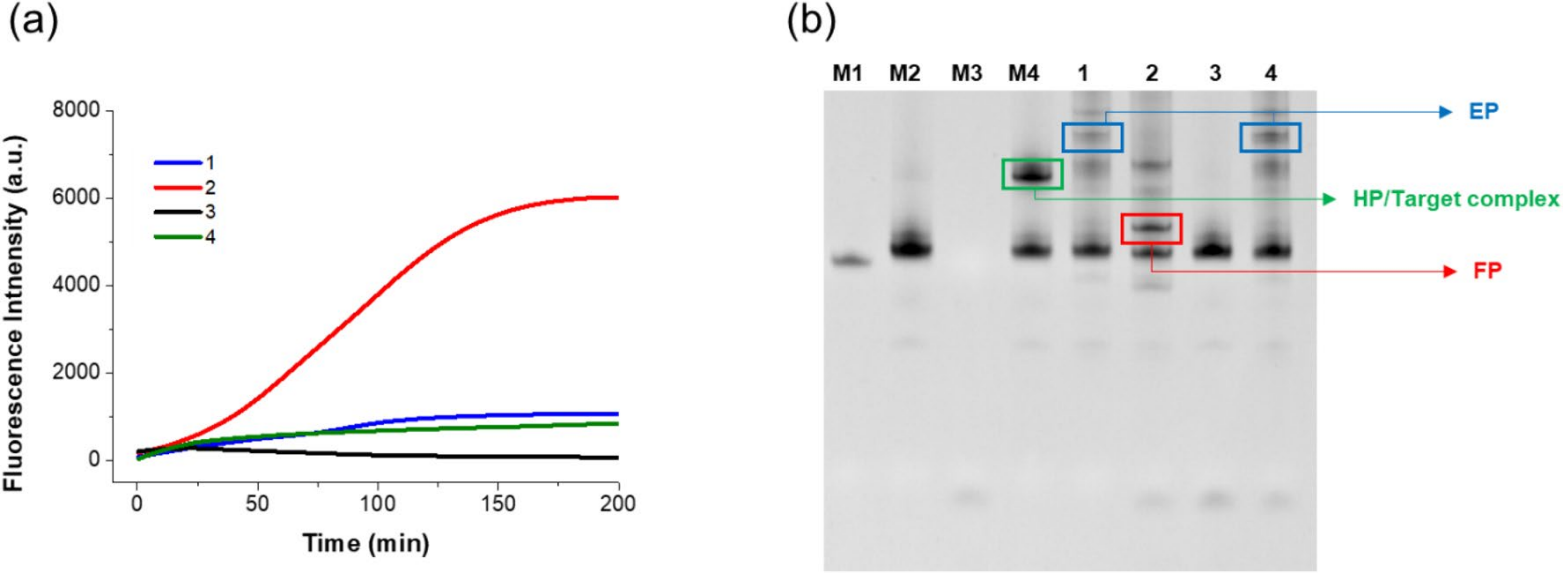
Feasibility of the PHAmp reaction. (**a**) Time-dependent fluorescence intensities produced from SYBR Green I staining during the PHAmp reaction (1: HP + Target DNA + Polymerase, 2: HP + Target DNA + Trigger + Polymerase, 3: HP + Trigger + Polymerase, and 4: Negative control HP + Target DNA + Trigger + Polymerase). The final concentrations of HP, trigger, polymerase, and target DNA are 50 nM, 1 μM, 0.125 U/μL, and 20 nM, respectively. (**b**) Polyacrylamide gel electrophoresis image of the PHAmp products (M1: Target DNA, M2: HP, M3: Trigger, M4: HP + Target DNA, 1: HP + Target DNA + Polymerase, 2: HP + Target DNA + Trigger + Polymerase, 3: HP + Trigger + Polymerase, and 4: Negative control HP + Target DNA + Trigger + Polymerase). In the PAGE analysis, the final concentrations of HP, trigger, polymerase, and target DNA are 500 nM, 1 μM, 0.125 U/μL, and 200 nM, respectively.

To validate the feasibility of the PHAmp reaction, we conducted the PHAmp reactions for synthetic 59-mer target ssDNA under various combinations of the reaction components and measured the time-dependent fluorescent signals produced from SYBR Green I staining for the reaction products. As presented in Fig. 2a, the rapid enhancement of the fluorescence signal was obviously observed only from the sample containing all reaction components which include HP, trigger, and DNA polymerase with target DNA (curve 2). In contrast, the fluorescence signal was negligible when the target DNA was omitted from the reaction sample (curve 3). When the trigger essential to the initiation and operation of the phase 2 reaction was excluded from the reaction sample, the fluorescent signal increased very slightly but was still quite low even in the presence of the target DNA. The slight increase in signal can be ascribed to the accumulation of EP produced by the phase 1 reaction, which is promoted only through the binding of target DNA to HP, but does not require the trigger strand. To verify that the PS modification at the 5′ overhang of HP is essential for the phase 2 reaction, a negative control HP with the same base sequence but without any PS modification at the 5′ overhang was examined for its capacity to actuate the PHAmp reaction. The negative control HP is capable of producing an EP through binding to the target DNA, but it is not able to initiate the phase 2 reaction because the stem end of the EP produced from the negative control HP without PS modification is just composed of normal DNA/DNA duplex and would not be readily denatured under the reaction temperatures. Therefore, there should be no trigger annealing site available within the EP. As expected, the fluorescence enhancement was quite negligible in this case (curve 4) and was almost the same as that seen in the absence of a trigger (curve 1). The above results confirm that the PHAmp reaction is only initiated when the target DNA is present, and the PS modification at the 5′ overhang of HP is critical to actuate the PHAmp reaction by destabilizing the PS-DNA/DNA duplexes within EP and IP and allowing the trigger to anneal to the EP and IP followed by the strand displacement DNA synthesis.

We next conducted polyacrylamide gel electrophoresis (PAGE) analysis for the products obtained from the PHAmp reactions to further support the fluorescence results. As shown in Fig. 2b, only lane 2, which contains all reaction components, showed an intense band corresponding to the FP of the PHAmp reaction. In contrast, only the applied HP band was observed in the absence of target DNA (lane 3). When the trigger was excluded or HP was replaced with a negative control HP, a strong band corresponding to the phase 1 product, EP, but no band for the phase 2 product, FP, was observed (lanes 1 and 4). These PAGE results strongly support the real-time fluorescence results.

We also performed melting curve analysis to obtain the further evidence for the formation of the reaction products during the PHAmp reaction. As shown in Figure S1, a clear peak corresponding to the melting point of FP (89.5 °C) was observed only from the sample containing all reaction components (curve 4), whereas only the melting point peak for unreacted HP (81 °C) was observed in the absence of target DNA (curve 5). A peak corresponding to the melting point of the phase 1 product, EP (88.5 °C), was also correctly observed when the trigger was excluded or HP was replaced with negative control HP (curves 3 and 6). All these results are quite consistent with the observations from the real-time fluorescence and PAGE analysis, again ensuring that the proposed PHAmp reaction operates according to the mechanism envisioned in Fig. 1.

### Optimization of the PHAmp reaction

The PHAmp reaction employs an HP with a PS modification at the 5′ overhang, and the length of the PS-DNA bases within HP should greatly influence the overall performance of the PHAmp reaction because the PS modification is exclusively responsible for initiating and operating the phase 2 reaction. To examine the effect of the length of the PS-DNA bases on the melting temperature, we first conducted melting curve analysis for PS-DNA/DNA duplexes with several selected lengths, which were then compared with the corresponding DNA/DNA duplexes. As shown in Figure S2, the melting temperatures of the PS-DNA/DNA duplexes were somewhat lower than those of the DNA/DNA duplexes in all cases, due to the reduced thermal stability caused by the PS modifications. They were estimated to be all higher than 55 °C and slightly increased as the length of the PS-DNA bases increased from 13 to 20. We envisioned that the trigger strand composed of normal bases could anneal to the stem end of EP and IP by replacing the less stable PS-DNA strand during spontaneous local conformational fluctuations within dsDNA although the melting temperatures of PS-DNA/DNA duplexes are still higher than the reaction temperature (37 °C). This DNA breathing phenomenon has been very effectively utilized to achieve several key isothermal amplification methods^31,33,34^.

We next conducted the PHAmp reactions by employing the HPs having different numbers of PS modification at the 5′ overhang and examined the time-dependent fluorescent intensities produced from the reaction products. As a result shown in Figure S3, the HP with 15 PS modifications solely resulted in the drastic fluorescence enhancement while the other HPs did not show any significant target-specific signal. We would interpret these results as follows: The HP_(13)_ having short 13 PS-DNA modifications should accompany the same short Trigger_(13)_, whose annealing on either EP or IP is not sufficiently stable to allow efficient extension in the following processes. On the other hand, the HP_(17)_ and HP_(20)_ having longer PS-DNA modifications should lead to the more stable PS-DNA/DNA duplex at the stem end due to the increased number of hybridized bases, which would greatly reduce the probability for the trigger to anneal to the stem end of EP and IP during dsDNA breathing dynamics and initiate the phase 2 reaction. Based on these results, the HP_(15)_ was employed as an optimal HP in all experiments of this work.

We also investigated the optimum reaction conditions, including the concentrations of HP, trigger, and polymerase as well as the reaction temperature by comparing the time-dependent fluorescence intensities of the reaction mixtures with target DNA to those without target DNA. The results showed that the following combination was optimal: 25 nM HP, 1 μM trigger, 0.125 U/μL DNA polymerase and a 37 °C incubation temperature (Figure S4, S5, S6 and S7). Thus, these conditions were used in further experiments.

### Sensitivity of the PHAmp reaction

**Figure 3:**
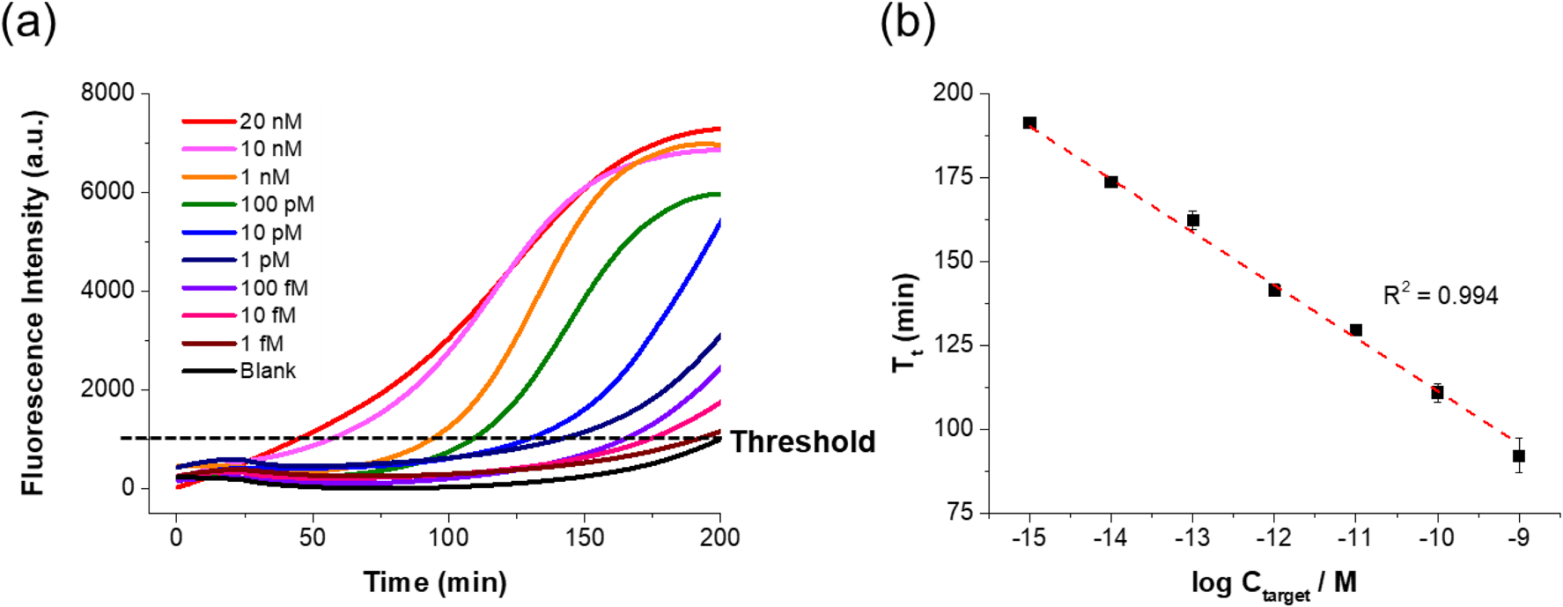
Sensitivity of the PHAmp reaction. (**a**) Time-dependent fluorescence intensities (via SYBR Green I staining) during the PHAmp reaction with target DNA at various concentrations. (**b**) Linear relationship between Tt and the logarithm of the target DNA concentration in the range from 1fM to 1 nM, where Tt is defined as the time at which the fluorescence signal reaches the threshold intensity (1000 a.u.) and C_target_ is the target DNA concentration. The error bars represent the standard deviations of three replicate measurements.

The sensitivity of the PHAmp reaction was determined by conducting the PHAmp reactions for target DNAs at a series of concentrations and measuring the real-time fluorescence signals from the reaction products. As shown in Fig. 3a, the threshold time (T_t_), defined as the time when the fluorescence signal reaches the threshold intensity (1000 a.u.), decreased gradually as the target DNA concentration increased in the range from 1 fM to 1 nM. When the T_t_ values were plotted as a function of the logarithm (log) of the target DNA concentration (Fig. 3b), an excellent relationship (T_t_ =  − 15.75 log (C_target/_M) + 190.25, R^2^ = 0.994) was obtained, confirming that the PHAmp reaction is quite capable of quantitatively identifying target DNA. The limit of detection (LOD = 3σ/S, where σ is the standard deviation of blank and S is the slope of the calibration curve) was estimated to be 0.29 fM, which is superior to previous alternative methods of isothermal amplification (Table S2).

### Specificity of the PHAmp reaction

The specificity of the PHAmp reaction was assessed by employing several nonspecific DNAs, including one- (MT 1), two- (MT 2), and three-base mismatched DNA (MT 3) and non-complementary DNAs (NC1 and NC2). The NC1 has non-complementary sequence within the entire strand, whereas NC2 has random sequence only at the HP binding site. We first predicted the ΔG values for HP and its complexes with specific or nonspecific target strands by using the OligoAnalyzer tool provided by IDT (Coralville, IA, USA). The results presented in Table S3 show that the ΔG is the most negative for HP/Target complex and its negative value decreased as the number of the mismatched bases increased from one to three as expected, indicating that the incorporation of the mismatched bases to target molecules destabilizes the formation of their complexes with HP^35^.

**Figure 4:**
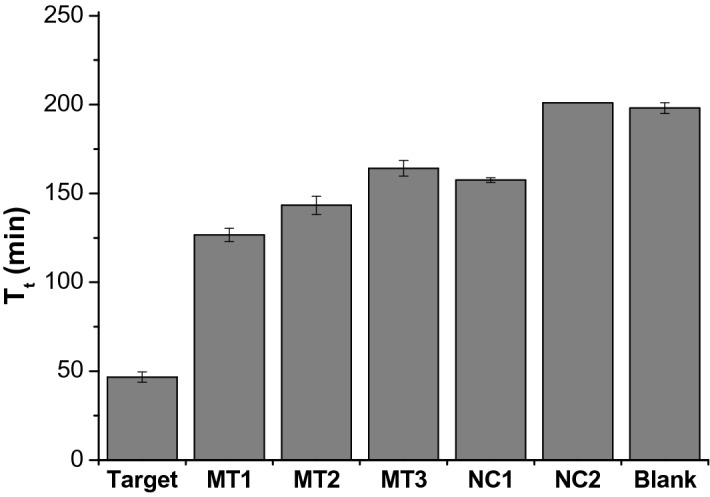
Specificity of the PHAmp reaction. The T_t_ values for target DNA and nonspecific target DNAs including several base-mismatched DNAs (MT1, MT2, and MT3) and non-complementary DNAs (NC1 and NC2) were determined and compared. T_t_ is defined as the time when the fluorescence signal reaches the threshold intensity (1000 a.u.).

The experimental data presented in Fig. 4 confirms that the T_t_ value from the target DNA is much smaller than those from the nonspecific targets such that the target DNA could be very reliably discriminated even from the only single base-mismatched target DNA. We might attribute this high specificity to the intrinsic mechanism of PHAmp reaction based on the stable secondary HP structure. More specifically the PHAmp reaction is not initiated only by the hybridization of target with HP but the hybridization affinity should be strong enough to fully open the hairpin structure of HP^36,37^. We assume that slightly weaker interaction of several base-mismatched target DNA with HP could lead to the much more intensified influence on the final signals, as evidenced in Fig. 4.

### Practical applicability of the PHAmp reaction

**Figure 5:**
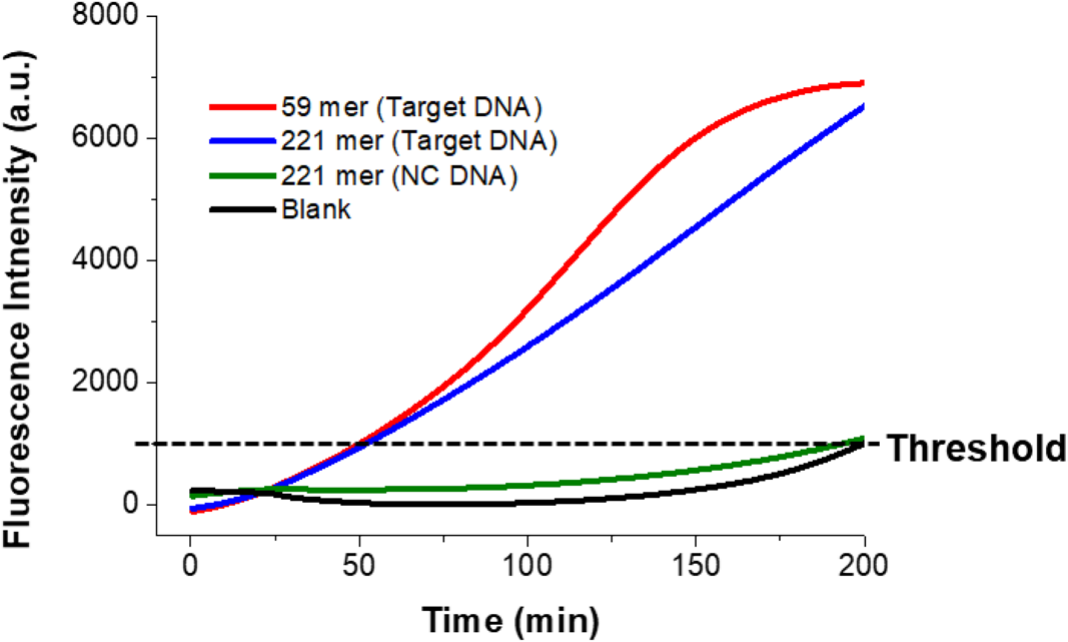
Practical utility test. Time-dependent fluorescence intensities (via SYBR Green I staining) during the PHAmp reaction in the presence of target DNA of different lengths. 221 bp DNAs were obtained from asymmetric PCR and the final concentration of the target DNAs was 20 nM.

We verified a practical diagnostic capability of the PHAmp reaction to reliably detect relatively long target nucleic acids which were prepared by asymmetric PCR of *Neisseria gonorrhoeae* plasmid DNA (target DNA) and *Leptospira interrogans* genomic DNA (NC DNA) followed by gel-based purification of ssDNA. It should be noted that this technique was developed to identify ss target nucleic acids as manifested in Fig. 1 but we conducted asymmetric PCR just to prepare relatively long ss target nucleic acids. As presented in Fig. 5, the 221-mer long target DNA was also successfully detected, showing almost the same T_t_ values with that from the synthetic 59-mer target DNA whereas the NC DNA produced very negligible fluorescence signal much like the blank sample without any target. These observations verify that the PHAmp method is capable of identifying target nucleic acids without limitation for their lengths.

To further evaluate the practical applicability of this strategy for complex heterogeneous biological samples, the PHAmp reaction was carried out for target DNAs in diluted human serum. As shown in Figure S9, the concentration of target DNAs spiked in the human serum also showed an excellent linear relationship with T_t_ values (T_t_ =  − 11.425 log (C_target/_M) + 9.2451, R^2^ = 0.992). Based on this linear relationship, the recovery tests were performed using target DNAs at three different concentrations. Table 1 shows that target DNA concentrations were very reliably determined in human serum with high reproducibility and precision as evidenced by the recovery rates between 92.01 and 115.04%. These results suggest that the proposed method could reliably determine target nucleic acids even in complex heterogeneous biological samples containing several interfering agents^38–41^.

**Table 1:** Recovery test for synthetic 59-mer target DNA spiked into diluted human serum.

Added concentration	Measured concentration^a^	SD^b^	CV (%)^c^	Recovery rate (%)^d^
100 pM	99.90 pM	2	8.69	99.90
10 pM	9.20 pM	1.15	12.50	92.01
10 fM	11.50 fM	1	2.00	115.04

^a^Mean of three measurements.

^b^Standard deviation of three measurements.

^c^Coefficient of variation = SD/mean × 100.

^d^Measured value/added value × 100.

## Conclusions

We developed a novel isothermal amplification method to detect target nucleic acids by designing a novel HP capable of initiating and operating a PHAmp reaction by binding to the target nucleic acid. The PHAmp reaction was able to successfully identify target nucleic acids down to 0.29 fM with an excellent specificity to discriminate even a single base-mismatched target. We also verified the practical applicability of the PHAmp reaction by demonstrating that it could identify long target without the limitation of the target length and target nucleic acids present in complex biological samples.

The PHAmp strategy described in this work offers several notable contributions to detection of target nucleic acids via isothermal amplification. First, by employing the HP ingeniously designed to contain a PS-DNA at the 5′ overhang and an SP region at the 3′ end, the PHAmp requires only one additional simple trigger strand and a single enzyme to accomplish the isothermal amplification, eliminating the requirements for multiple enzymes or complicated combinations of primers as in many other isothermal methods. Second, the PHAmp reaction is performed at a relatively low temperature of 37 °C, which would enhance the applicability of the method, especially for POC or on-site diagnostics, by reducing both device complexity and power consumption. Third, it relies on an intrinsic HP structure that opens only through complete binding of the target to the loop of the HP, consequently ensuring very high specificity toward the target nucleic acid.

This PHAmp strategy could serve as a powerful isothermal platform to reliably identify ss target nucleic acids, which include genomic RNAs from coronaviruses which cause Severe Acute Respiratory Syndrome (SARS)^42^, Middle East Respiratory Syndrome (MERS)^43^ and Coronavirus Disease 2019 (COVID-19)^34^, genomic ssDNAs from *Parvoviridae* B19^44^, minute virus of mice^45^ and adeno-associated virus (AAV)^46^, mRNAs and miRNAs.

## Methods

### Materials

All oligonucleotides used in this study were synthesized and purified using PAGE by Bioneer (Daejeon, South Korea), except for the trigger, which was purified by Bio-RP (Bioneer, Daejeon, South Korea). The oligonucleotide sequences are listed in Table S1. The Klenow fragment (3′ → 5′ exo-), 10X NEBuffer 2 (100 mM Tris–HCl, pH 7.9, 500 mM NaCl, 100 mM MgCl_2_, and 10 mM DTT), and deoxynucleotide solution mixture (dNTPs) were purchased from New England Biolabs, Inc. (Beverly, MA, USA). SYBR Green I (10,000X) was purchased from Invitrogen (Carlsbad, CA, USA). Leptospira interrogans genomic DNA was purchased from ATCC (Manassas, VA, USA). Human serum was purchased from Sigma-Aldrich (St. Louis, MO, USA). Ultrapure DNase/RNase-free distilled water (DW) was purchased from Bioneer (Daejeon, South Korea) and used in all experiments. All other chemicals were of analytical grade and used without further purification.

### PHAmp reaction for target DNA detection

HP (1 μM in 1X NEBuffer 2) was heated at 90 °C for 5 min, gradually cooled to 25 °C, and incubated at 4 °C before use. The PHAmp reaction was conducted in a 20 μL reaction volume containing 25 nM HP, 1 μM trigger, 250 μM dNTPs, 1X SYBR Green I, 0.125 U/μL Klenow fragment, and target DNA at various concentrations in 1X NEBuffer 2. The reaction solution was incubated at 37 °C, and the fluorescence signal resulting from SYBR Green I staining was measured every 30 s on a CFX Connect Real-Time PCR Detection System (Bio-Rad, CA, USA).

For the detection of nucleic acids in complex heterogeneous biological samples, human serum samples spiked by target DNA were employed as target. The final concentration of diluted human serum was 1%.

### Polyacrylamide gel electrophoresis (PAGE) analysis

The reaction mixtures were prepared according to the “PHAmp reaction for target DNA detection” guidelines, except that 500 nM HP was used instead of 25 nM HP and 1X SYBR Green I was excluded. After incubation at 37 °C for 60 min, a 10 μL aliquot of the reaction solution was resolved on a 15% polyacrylamide gel. Electrophoresis was carried out at a constant voltage of 120 V for 120 min using 1X TBE as a running buffer. After staining with ethidium bromide (EtBr) (Bioneer, Daejeon, South Korea), an image of the gel was taken with a Gel Doc EZ Imager (Bio-Rad, CA, USA)^23,24,35^.

### Melting curve analysis

Reaction mixtures (20 μL) were prepared according to the “PHAmp reaction for target DNA detection” guidelines, except that 500 nM HP was used instead of 25 nM HP. After incubation at 37 °C for 120 min, the fluorescence signal was measured on a CFX Connect Real-Time PCR Detection System (Bio-Rad, CA, USA) as the temperature was increased from 20 °C to 95 °C in an increment of 0.5 °C. The melting temperature was determined based on the first derivative plot [−d(RFU)/dT]^23^.

### Preparation of 221-mer target DNA

Normal PCR was conducted in a 20 μL reaction mixture containing 10^5^ copies of template DNA, 250 μM dNTPs, 0.05 U/μL i-Taq DNA polymerase, 500 nM forward primer, and 500 nM reverse primer in 1X PCR reaction buffer (10 mM Tris–HCl, pH 8.3, 50 mM KCl, and 2 mM MgCl_2_). For PCR amplification, the reaction mixtures were heated to 95 °C for 5 min, followed by 40 cycles of 95 °C for 30 s, 55 °C for 30 s, and 72 °C for 30 s, and finalized at 72 °C for 5 min^23,24,35^. Previously prepared *Neisseria gonorrhoeae* plasmid DNA (target DNA)^47^ and *Leptospira interrogans* genomic DNA (non-complementary (NC) DNA) were employed as template DNAs for amplification. To obtain 221-mer ssDNA, asymmetric PCR was conducted by following the same procedures but using different forward and reverse primer concentration ratios (5:1, 10:1, and 20:1) (Figure S8).

After completion of the reaction, a 5 μL aliquot of the PCR products was resolved on a 3% agarose gel containing EtBr, and electrophoresis was carried out at a constant voltage of 100 V for 40 min using 1X TBE as a running buffer. After gel electrophoresis, an image of the gel was taken with a Gel Doc EZ Imager (Bio-Rad, CA, USA). The PCR products were purified using a NucleoSpin Gel and PCR clean-up kit (Macherey–Nagel, Düren, Germany), and the concentrations of the purified products were measured using a Nanodrop One spectrophotometer (Thermo Scientific, MA, USA).

## Supplementary Information

Supplementary Information.
