# Supplementary material for: Ultrasensitive nucleic acid detection based on phosphorothioated hairpin-assisted isothermal amplification
Source: Sci Rep. 2021 Apr 16;11:8399. doi: 10.1038/s41598-021-87948-8 (PMC8052315; doi:10.1038/s41598-021-87948-8)
Supplement: Supplementary file 1 — Supplementary Information. [file 41598_2021_87948_MOESM1_ESM.docx]

Supporting Information for

**Ultrasensitive nucleic acid detection based on phosphorothioated hairpin-assisted isothermal amplification**

Yujin Jung,^a†^ Jayeon Song,^a†^ and Hyun Gyu Park^a^*

^a^ Department of Chemical and Biomolecular Engineering (BK 21+ program),

KAIST, Daehak-ro 291, Yuseong-gu, Daejeon 34141, Republic of Korea

*To whom correspondence should be addressed.

E-mail: hgpark@kaist.ac.kr (H.G. Park); Phone: +82-42-350-3932; Fax: +82-42-350-3910.

^†^These authors equally contributed to this work.

**Table S1.** Oligonucleotide sequences studied in this work.

| **Name** | **DNA sequence (5’ → 3’)^a^** |
| --- | --- |
| **Target DNA^b^** | AGG TCT AG**G GTG CGC TCT GCT TCG GCT CT**C TGC TGT TTC AAG TCG TCC AGC TCG TTC TT |
| **MT1^b, c^** | AGG TCT AG**G GTC CGC TCT GCT TCG GCT CT**C TGC TGT TTC AAG TCG TCC AGC TCG TTC TT |
| **MT2 ^b, c^** | AGG TCT AG**G GTC GGC TCT GCT TCG GCT CT**C TGC TGT TTC AAG TCG TCC AGC TCG TTC TT |
| **MT3 ^b, c^** | AGG TCT AG**G GTC GCC TCT GCT TCG GCT CT**C TGC TGT TTC AAG TCG TCC AGC TCG TTC TT |
| **NC1** | AGC TTT GGC GAT TTG GTC AGG CAT AAT CGC CGA CAT TCT TTC TAC ACG GAT CCA AGT AT |
| **NC2** | AGG TCT AG GAA TTC G AGA CGC ATA ATC GCC TGC TGT TTC AAG TCG TCC AGC TCG TTC TT |
| **HP_(13)_^d^** | ATG CGC CCT ACT C CTG GAC GAC TTG AAA CAG CAG **AGA GCC GAA GCA GAG CGC ACC** CTG CTG TTT CAA GTC GTC CAG TTG AAA |
| **HP_(15)_^d^** | ATG CGC CCT ACT CAT CTG GAC GAC TTG AAA CAG CAG **AGA GCC GAA GCA GAG CGC ACC** CTG CTG TTT CAA GTC GTC CAG TTG AAA |
| **HP_(17)_^d^** | ATG CGC CCT ACT CAT AC CTG GAC GAC TTG AAA CAG CAG **AGA GCC GAA GCA GAG CGC ACC** CTG CTG TTT CAA GTC GTC CAG TTG AAA |
| **HP_(20)_^d^** | ATG CGC CCT ACT CAT ACT AT CTG GAC GAC TTG AAA CAG CAG **AGA GCC GAA GCA GAG CGC ACC** CTG CTG TTT CAA GTC GTC CAG TTG AAA |
| **Trigger_(13)_^e^** | ATG CGC CCT ACT C |
| **Trigger_(15)_^e^** | ATG CGC CCT ACT CAT |
| **Trigger_(17)_^e^** | ATG CGC CCT ACT CAT AC |
| **Trigger_(20)_^e^** | ATG CGC CCT ACT CAT ACT AT |
| **Forward primer** | TTG GTT TCC AAC AGG TCT AGG G |
| **Reverse primer** | CAA AGC GAG CAG AAA ATA ACC G |
| **Forward primer (NC)** | CAA AGA CTC TTC AGC AGC GAT A |
| **Reverse primer (NC)** | CAA AAT CTT TTG ACG ATC TGA AA |
| ^a^ The colors of oligonucleotide sequences correspond to those of the domains depicted in Scheme 1.  ^b^ Bold letters in target DNA and mismatched DNAs (MTs) represent the sequence complementary to HP.  ^c^ Underlined letters in MTs indicate the bases mismatched with target recognition site in HP.  ^d^ Green, red, and purple colored letters in HPs represent PS-DNA overhang, stem region of SP region, and loop region of SP region, respectively.  ^d^ Bold letters in HPs represent the target recognition site.  ^d^ The number in the bracket indicates the base length of the PS-DNA overhang.  ^e^ The number in the bracket indicates the length of the trigger. | |

**Table S2.** Comparison of the developed method with previous isothermal amplification methods for target nucleic acid detection.

| **Key materials**  **or**  **methods** | **Linear range** | **LOD** | **Limitations** | **Reference** |
| --- | --- | --- | --- | --- |
| Two hairpin drive-based DNA nanomachine | 1 pM – 8 nM | 1 pM | - Modification of fluorophore and quencher  - Requirement of two enzymes | ^1^ |
| Aligner-mediated cleavage | 1 fM – 1 nM | 1 fM | - Requirement of two enzymes | ^2^ |
| Cascade enzymatic signal amplification | 1 fM – 10 pM | 1 fM | - Modification of fluorophore and quencher  - Requirement of three enzymes | ^3^ |
| Exponential rolling circle amplification | 100 pM – 40 nM | 1 pM | - Modification of fluorophore and quencher  - Requirement of two enzymes | ^4^ |
| Cascade circular exponential amplification | 1 fM – 100 pM | 0.61 fM | - Modification of fluorophore and quencher  - Requirement of two enzymes | ^5^ |
| Single palindromic molecular beacon-based amplification | 100 pM – 75 nM | 100 pM | - Modification of fluorophore and quencher | ^6^ |
| PHAmp | 1 fM – 1 nM | 0.29 fM | - | This work |

| **Structure** | **ΔG (kcal/mole)** |
| --- | --- |
| HP | -29.73 |
| HP/Target complex | -45.83 |
| HP/MT1 complex | -36.33 |
| HP/MT2 complex | -32.71 |
| HP/MT3 complex | -29.58 |

**Table S3.** Predictive structure stability of HP, HP/Target complex, and HP/nonspecific target complexes. The thermodynamic calculation was performed by using the OligoAnalyzer tool provided by IDT (Coralville, IA, USA).

In the PHAmp reaction, the EP increases by one per each cycle, producing a total of n EPs as the n cycles of the PHAmp reaction progress. Since one EP can generate one FP for each cycle, the number of FPs produced at n cycle is n. These created FPs are remained and are not reused for the next cycle, therefore the number of FPs increases with the sigma function as shown below.

The number of FPs created after n cycles of PHAmp reaction =

$$\sum_{k=1}^{n} k= \frac{n(n+1)}{2}= \frac{1}{2} (n^{2}+n)$$
Figure S1. Melting curve analysis for the PHAmp reaction (1: HP, 2: HP + Target DNA, 3: HP + Target DNA + Polymerase, 4: HP + Target DNA + Trigger + Polymerase, 5: HP + Trigger + Polymerase, and 6: Negative control HP + Target DNA + Trigger + Polymerase). The final concentrations of HP, trigger, polymerase, and target DNA are 500 nM, 1 μM, 0.125 U/μL, and 200 nM, respectively.


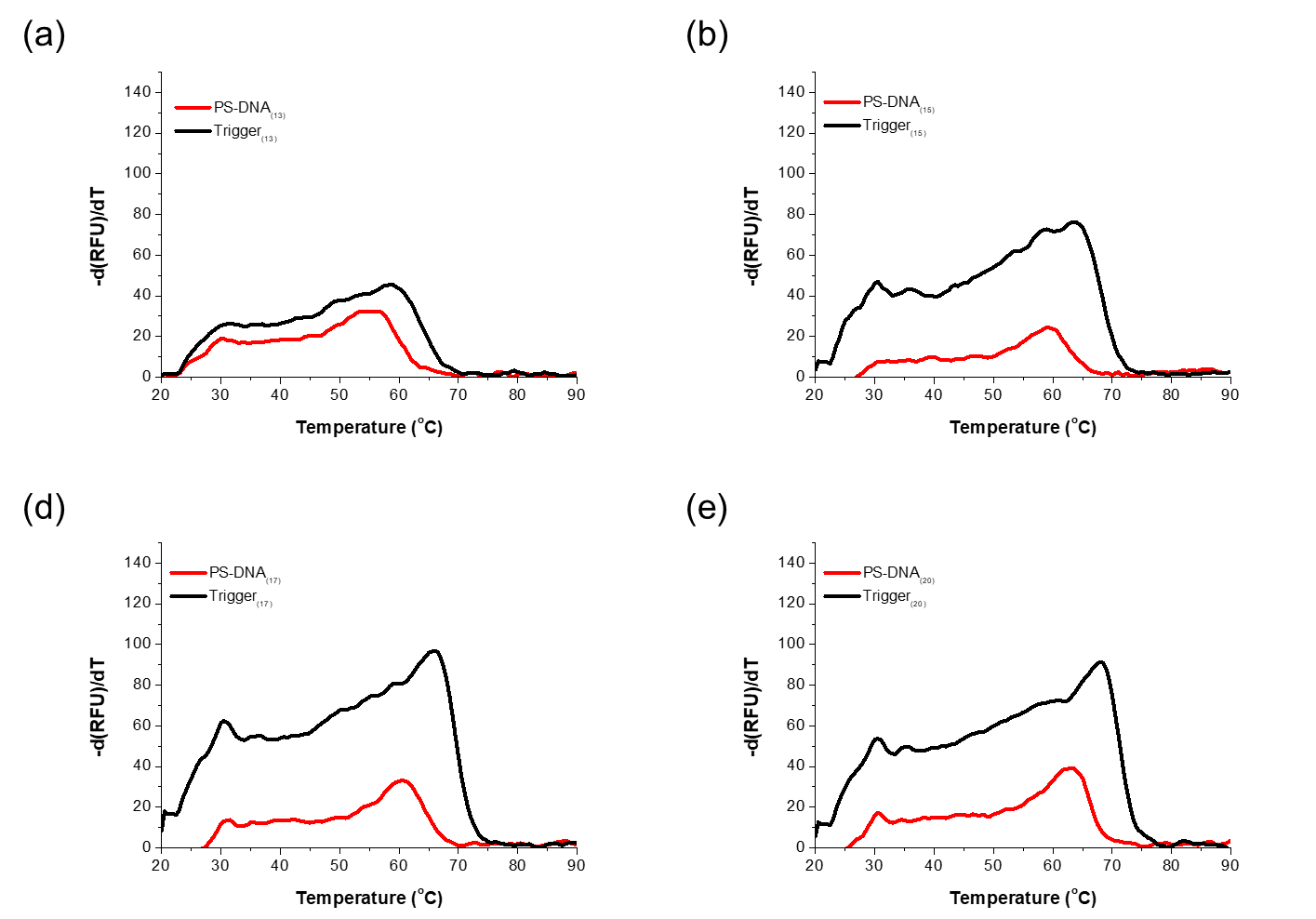


Figure S2. Melting curve analysis for the comparison of PS-DNA/DNA and DNA/DNA duplex with the length of (a) 13mer, (b) 15mer, (c) 17mer, (d) 20mer. (Red: PS-DNA/DNA duplex, Black: DNA/DNA duplex) The sequences of PS-DNAs are identical with those of trigger strands in Table S1 and the opposite DNAs are their complementary DNAs. The final concentrations of PS-DNA, trigger, and complement DNA are 1 μM.


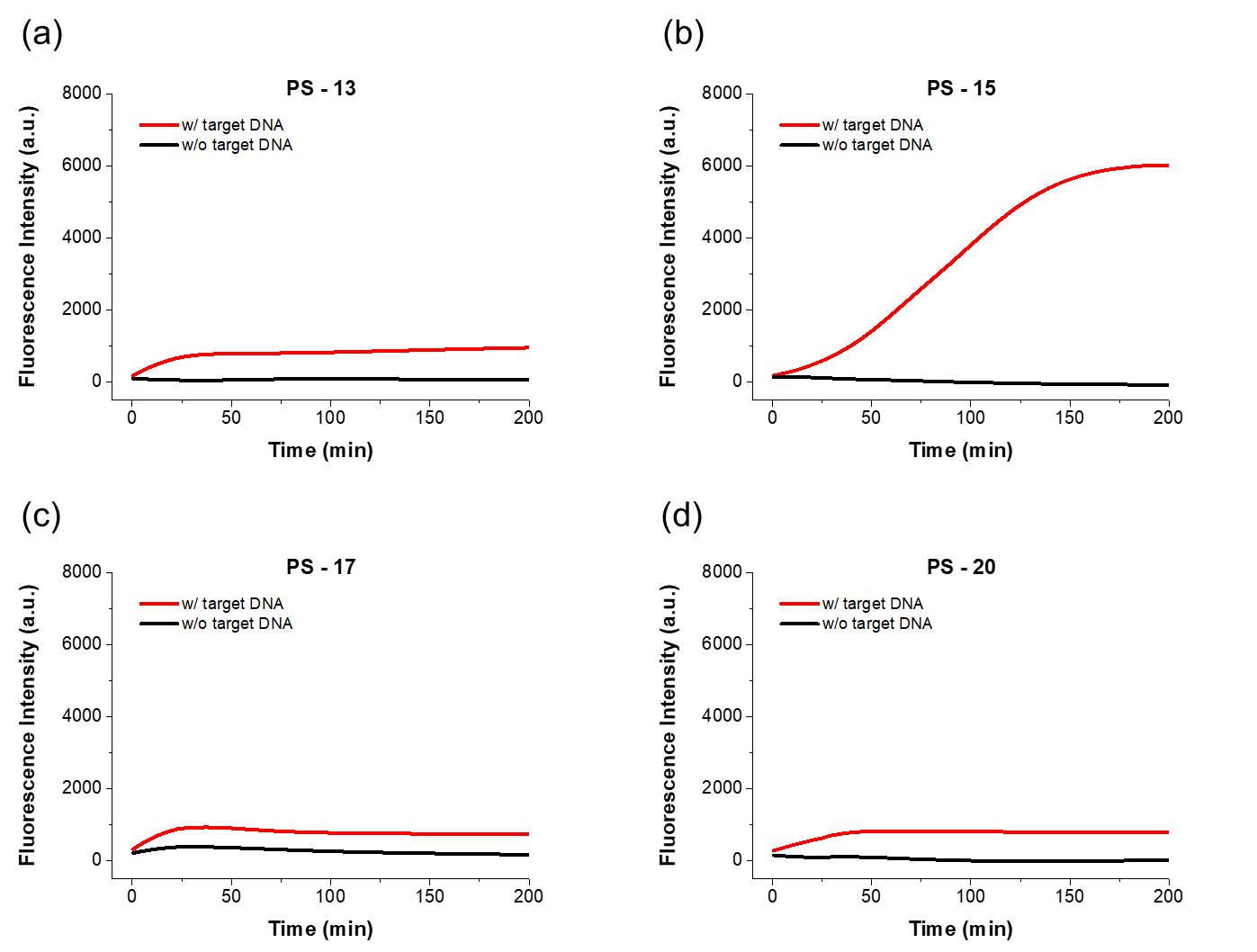
**Figure S3.** Effect of the number of PS modifications at 5’ overhang of HP. Time-dependent fluorescence intensities produced from SYBR Green I staining during the PHAmp reaction in the presence (Red) and absence of target DNA (Black). The number of PS modifications are (a) 13, (b) 15, (c) 17, and (d) 20. The final concentrations of HP, trigger, polymerase, and target DNA are 50 nM, 1 μM, 0.125 U/μL, and 20 nM, respectively.


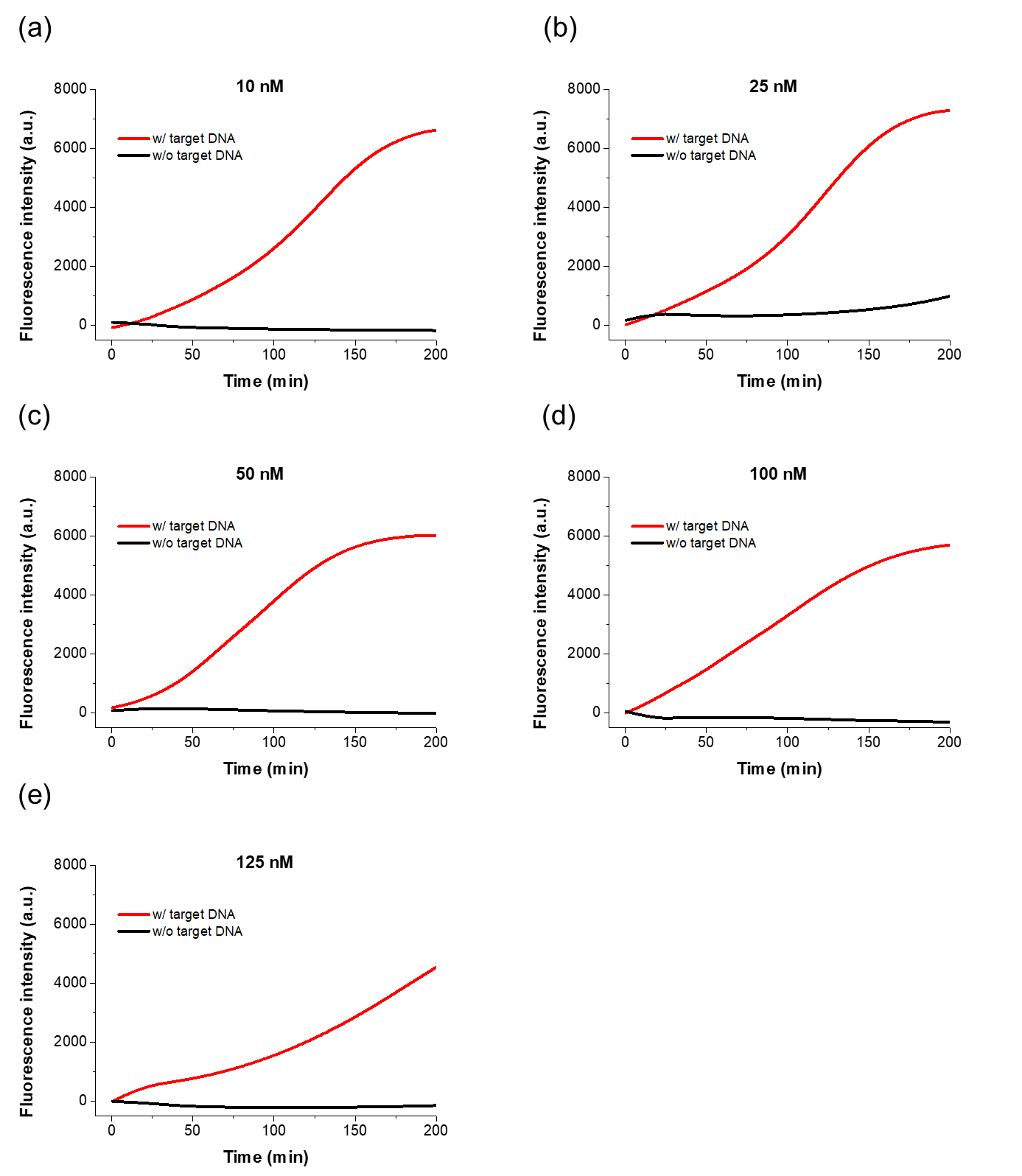
Figure S4. Optimization of the HP concentration. Time-dependent fluorescence intensities produced from SYBR Green I staining during the PHAmp reaction in the presence (Red) and absence of target DNA (Black). The concentrations of HP are (a) 10 nM, (b) 25 nM, (c) 50 nM, (d) 100 nM, and (e) 125 nM. The final concentrations of trigger, polymerase, and target DNA are 1 μM, 0.125 U/μL, and 20 nM, respectively.


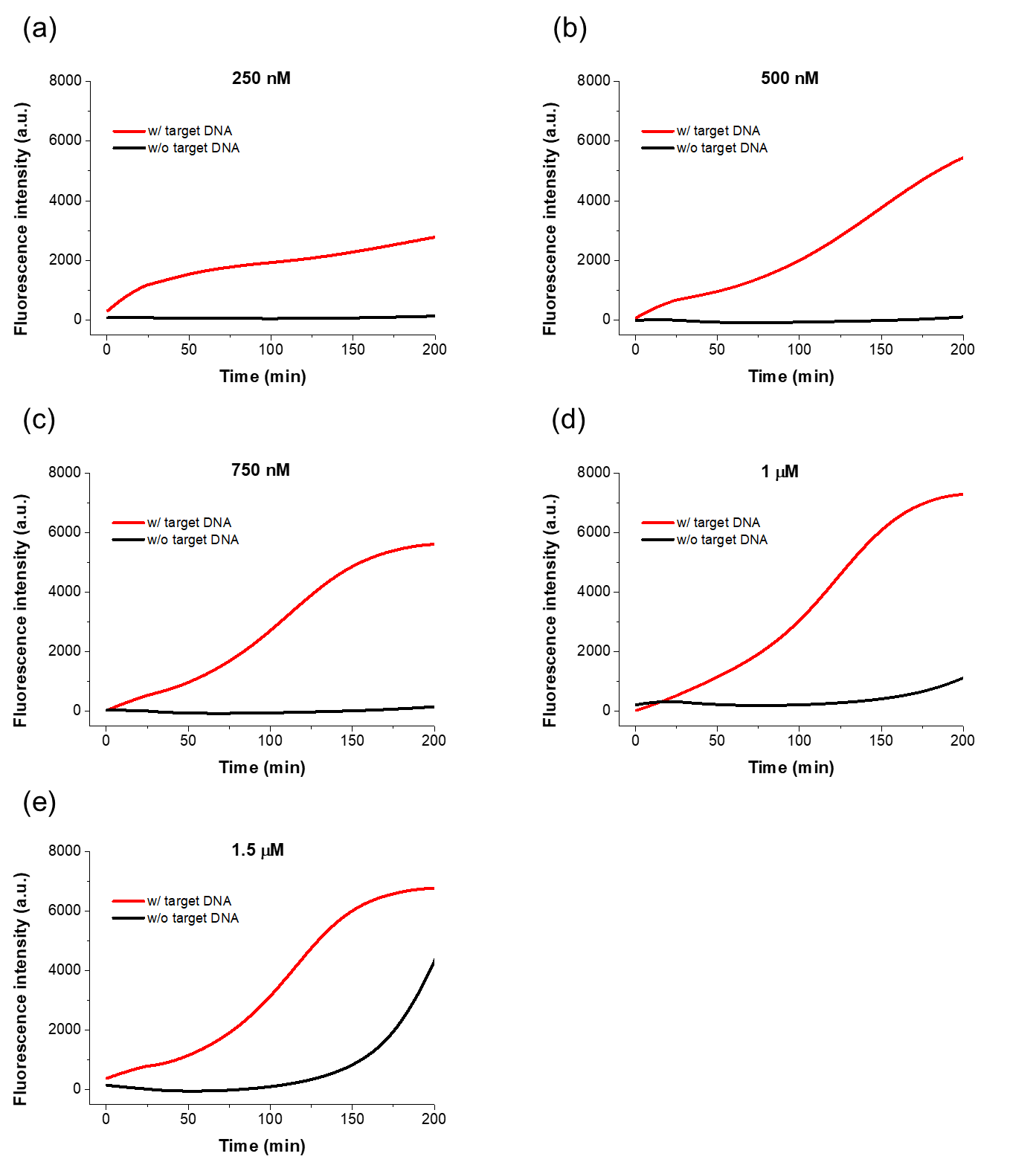
Figure S5. Optimization of the trigger concentration. Time-dependent fluorescence intensities produced from SYBR Green I staining during the PHAmp reaction in the presence (Red) and absence of target DNA (Black). The concentrations of trigger are (a) 250 nM, (b) 500 nM, (c) 750 nM, (d) 1 μM, and (e) 1.5 μM. The final concentrations of HP, polymerase, and target DNA are 25 nM, 0.125 U/μL, and 20 nM, respectively.


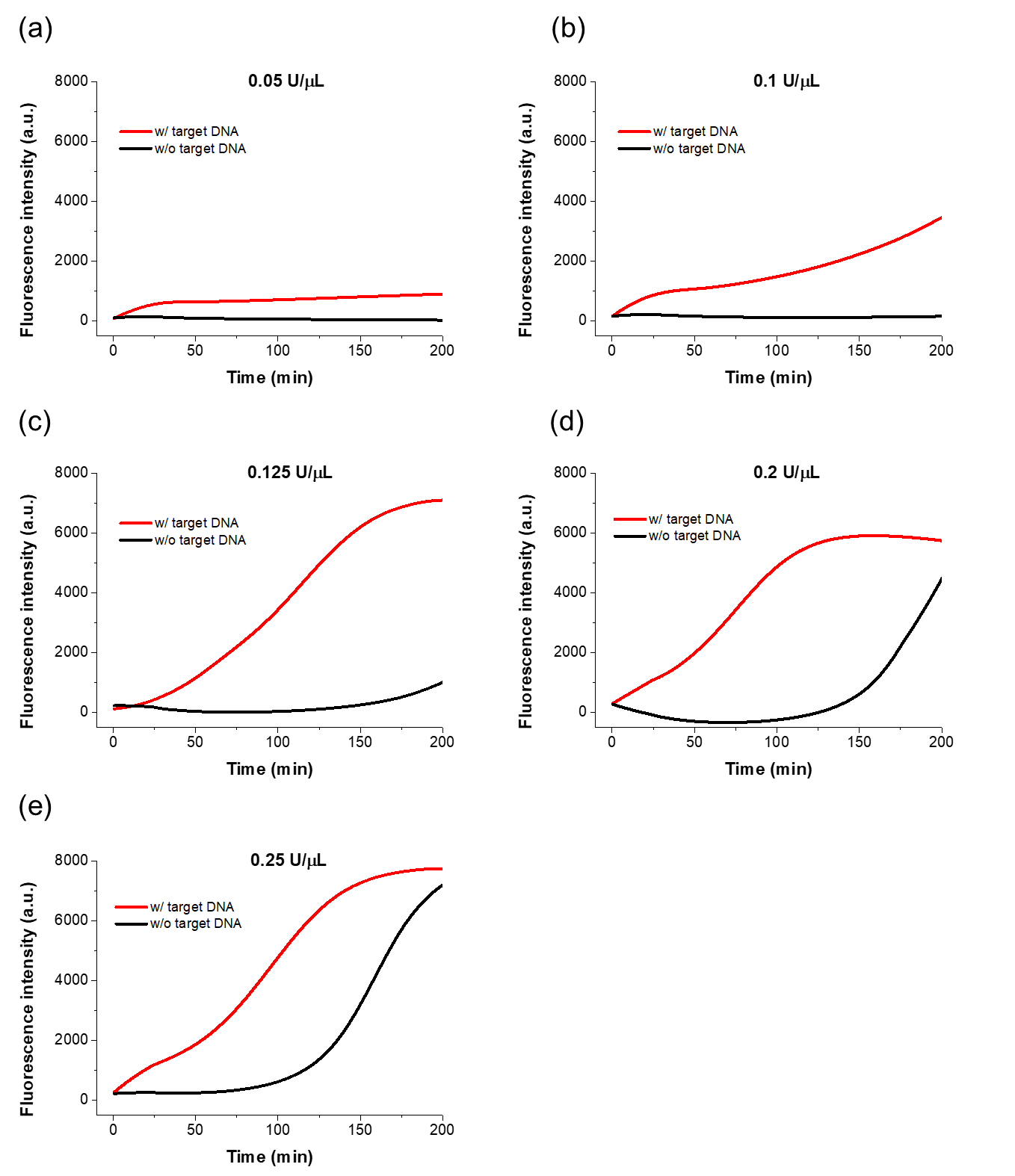
Figure S6. Optimization of the concentration of polymerase. Time-dependent fluorescence intensities produced from SYBR Green I staining during the PHAmp reaction in the presence (Red) and absence of target DNA (Black). The concentrations of polymerase are (a) 0.05 U/μL, (b) 0.1 U/μL, (c) 0.125 U/μL, (d) 0.2 U/μL, and (e) 0.25 U/μL. The final concentrations of HP, trigger, and target DNA are 25 nM, 1 μM, and 20 nM, respectively.


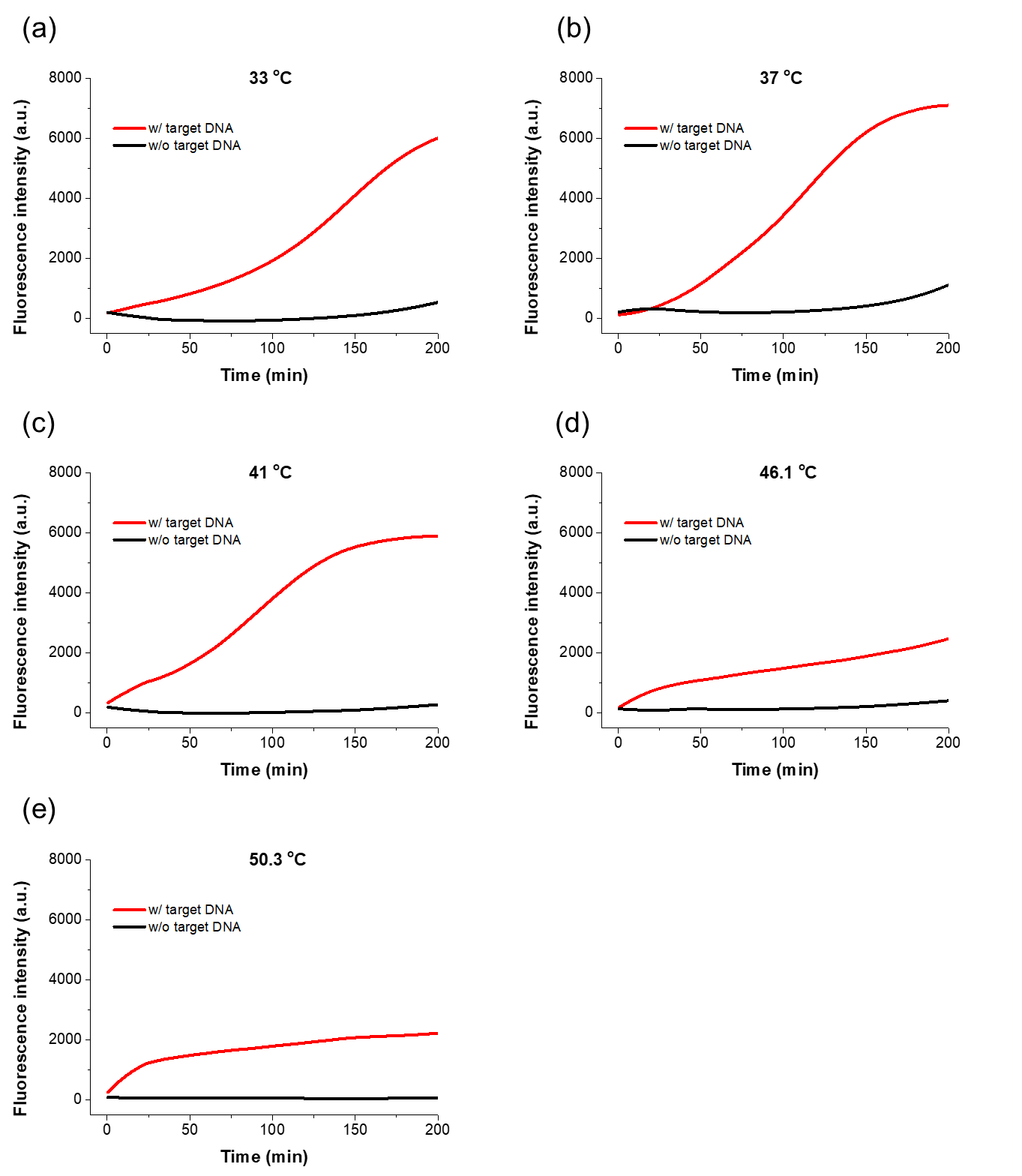
Figure S7. Optimization of the reaction temperature. Time-dependent fluorescence intensities produced from SYBR Green I staining during the PHAmp reaction in the presence (Red) and absence of target DNA (Black) at various reaction temperatures, which are (a) 33 °C, (b) 37 °C, (c) 41 °C, (d) 46.1 °C, and (e) 50.3 °C. The final concentrations of HP, trigger, polymerase, and target DNA are 25 nM, 1 μM, 0.125 U/μL, and 20 nM, respectively.


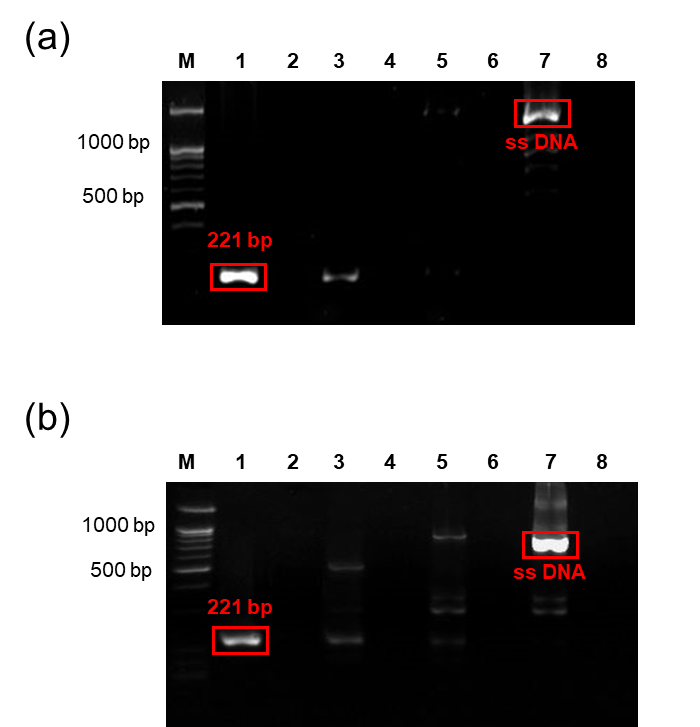
Figure S8. The agarose gel electrophoresis image of the PCR products obtained from (a) target DNA and (b) NC DNA by using various concentration ratios of forward primer to reverse primer (M: 100 bp DNA ladder, 1 and 2: Positive and negative samples, respectively, using 500 nM forward primer and 500 nM reverse primer (1:1 ratio), 3 and 4: Positive and negative samples, respectively, using 500 nM forward primer and 100 nM reverse primer (5:1 ratio), 5 and 6: Positive and negative samples, respectively, using 500 nM forward primer and 50 nM reverse primer (10:1 ratio), 7 and 8: Positive and negative samples, respectively, using 500 nM forward primer and 25 nM reverse primer (20:1 ratio)). Positive and negative samples indicate the samples obtained after PCR with and without template target DNA, respectively. The reaction products produced using 20:1 primer concentration ratio were subjected to gel purification of ss DNA, which were then employed in the practical utility test of the PHAmp reaction.

**Figure S9.** The correlation of T_t_ to the logarithm of the concentration of target DNAs spiked in diluted human serum (1%). The Tt was defined as the time when the fluorescence signal reaches the threshold intensity (1000 a.u.).

**References**

1 Xu, J. *et al.* Two-wheel drive-based DNA nanomachine and its sensing potential for highly sensitive analysis of cancer-related gene. *Biomaterials* **100**, 110-117 (2016).

2 Wu, W. *et al.* Aligner-mediated cleavage of nucleic acids and its application to isothermal exponential amplification. *Chem. Sci.* **9**, 3050-3055 (2018).

3 Zou, B., Ma, Y., Wu, H. & Zhou, G. Ultrasensitive DNA detection by cascade enzymatic signal amplification based on Afu flap endonuclease coupled with nicking endonuclease. *Angew. Chem. Int. Ed. Engl.* **123**, 7533-7536 (2011).

4 Xu, H. *et al.* Exponential rolling circle amplification and its sensing application for highly sensitive DNA detection of tumor suppressor gene. *Sens. Actuators B Chem.* **243**, 1240-1247 (2017).

5 Sun, X., Wang, L., Zhao, M., Zhao, C. & Liu, S. An autocatalytic DNA machine with autonomous target recycling and cascade circular exponential amplification for one-pot, isothermal and ultrasensitive nucleic acid detection. *Chem. Commun.* **52**, 11108-11111 (2016).

6 Li, F. *et al.* Single palindromic molecular beacon-based amplification for genetic analysis of cancers. *Biosens. Bioelectron.* **91**, 692-698 (2017).
